# Infection of Bacterial Endosymbionts in Insects: A Comparative Study of Two Techniques viz PCR and FISH for Detection and Localization of Symbionts in Whitefly, *Bemisia tabaci*

**DOI:** 10.1371/journal.pone.0136159

**Published:** 2015-08-19

**Authors:** Harpreet Singh Raina, Ambika Singh, Sonam Popli, Neeti Pandey, Raman Rajagopal

**Affiliations:** Gut Biology Laboratory, Department of Zoology, University of Delhi, Delhi, India; University of Minnesota, UNITED STATES

## Abstract

Bacterial endosymbionts have been associated with arthropods and large number of the insect species show interaction with such bacteria. Different approaches have been used to understand such symbiont- host interactions. The whitefly, *Bemisia tabaci*, a highly invasive agricultural pest, harbors as many as seven different bacterial endosymbionts. These bacterial endosymbionts are known to provide various nutritional, physiological, environmental and evolutionary benefits to its insect host. In this study, we have tried to compare two techniques, Polymerase chain reaction (PCR) and Flourescence *in situ* Hybridisation (FISH) commonly used for identification and localization of bacterial endosymbionts in *B*. *tabaci* as it harbors one of the highest numbers of endosymbionts which have helped it in becoming a successful global invasive agricultural pest. The amplified PCR products were observed as bands on agarose gel by electrophoresis while the FISH samples were mounted on slides and observed under confocal microscope. Analysis of results obtained by these two techniques revealed the advantages of FISH over PCR. On a short note, performing FISH, using LNA probes proved to be more sensitive and informative for identification as well as localization of bacterial endosymbionts in *B*. *tabaci* than relying on PCR. This study would help in designing more efficient experiments based on much reliable detection procedure and studying the role of endosymbionts in insects.

## Introduction

The term symbiosis refers to a permanent association between two or more distinct individuals, called symbionts, atleast during a part of their life cycle. The organisms which live inside the cell of the other are called endosymbionts. Symbiotic relationship can exist at various levels: between prokaryotes and eukaryotes, between unicellular and multicellular organisms etc. In fact, the symbiotic associations can be grouped as mutualism, commensalism, parasitism with respect to the effect of that symbiont on the host [1]. Symbiotic bacteria are ubiquitous in animal hosts and in some invertebrate hosts, they live an intracellular existence for much of their life and are vertically transmitted. It has been estimated that around 15% of all insects possess such bacterial endosymbionts [2,3]. Different insects like psyllids, aphids, mealybugs, whiteflies which belong to suborder sternorrhycha of order Hemiptera have been reported to have bacterial endosymbionts.

The insect endosymbionts have been categorized into Primary and Secondary endosymbionts. The Primary endosymbionts have an obligatory relationship with the insect host, providing essential aminoacids and showing phylogenetic congruence with their host [4,5]. The secondary endosymbionts have a facultative relationship and a short evolutionary history with their host [5,6]. These secondary endosymbionts are reported to perform a variety of functional roles on their hosts, such as providing fitness benefits [7], increasing tolerance to heat stress [8], increasing resistance to parasitic wasps [9], causing host plant specialization [10], conferring invasiveness [11]. In fact, several secondary endosymbionts appear to affect the capacity of the host to be a pest. Moreover, Clark et al. [12]; Gibson and Hunter [13]; Douglas [14] have discussed the role of different endosymbionts in insects. Hence, it is important to detect the different types of bacterial endosymbionts present in insects.

*Bemisia tabaci* (Gennadius) (Homoptera: Aleyrodidae) is a worldwide pest of agricultural, ornamental and field crops [15]. It is also known as cotton whitefly, cassava whitefly and tobacco whitefly. They are sap sucking insects and feed on the phloem sap of a wide range of vegetables like brinjal (egg plant), cabbage, raddish, tomato, beans, cucurbits, potato; cash crops like cotton, sunflower, tobacco; legumes etc. They damage the crops by directly feeding on them and indirectly by producing honeydew and vectoring 115 different pathogenic plant viruses [16]. *B*. *tabaci* also harbors both Primary endosymbionts and Secondary endosymbionts. *Portiera aleyrodidarum* is the only primary endosymbiont of the whitefly while secondary endosymbionts include a range of bacteria for example *Wolbachia* (Rickettsiales), *Arsenophonus* (Enterobacteriales), *Cardinium* (Bacteriodetes), *Rickettsia* (Rickettsiales), *Hamiltonella* (Enterobacteriales), *Fritschea* (Chlamydiales) [17]. Different genetic groups of *B*. *tabaci* have been described to be infected by different secondary endosymbiont combinations. These endosymbionts have been shown to be responsible for conferring important abilities to their host *B*. *tabaci*. *Hamiltonella* has been described to have a significant contribution in virus transmission to plants [18]. Similarly, it has been reported that *Arsenophonus* helps in transmission of cotton leaf curl virus [19]. A well documented role of *Rickettsia* in heat tolerance and increased susceptibility to some insecticides has been reported [20].

Therefore, in light of the compelling evidences of the role of the endosymbionts in *B*. *tabaci* and the benefits they provide to their host, the detailed functions of these endosymbionts should be studied. However, the basis of all these studies is the detection and identification of these bacterial endosymbionts. Many techniques have been used for identification, detection and localization of these endosymbionts including electron microscopy, PCR, confocal microscopy (FISH- Flourescence *in situ* Hybridization) etc. Costa et al. [21] examined the ultrastructure, morphology and the frequency of endosymbionts of *B*. *tabaci* in different geographic regions using electron microscopy. But this technique determines and identifies the bacteria only on the basis of their morphology which is a limiting factor in terms of identification of endosymbionts. The other technique used for identification of endosymbionts is PCR with bacterial gene specific primers. Different gene targets like 16S, 23S, GroEL etc have been used for the identification of bacteria. Several studies have used PCR technique to identify the different endosymbionts like *Portiera*, *Wolbachia*, *Rickettsia*, *Arsenophonus*, *Cardinium*, *Hamiltonella*, *Fritschea in B*. *tabaci* [22,23,24,25,26,27]. In fact, this technique is widely used for the detection and identification of endosymbionts in most of the insect hosts. But, unfortunately it is not able to provide any information on the localization of endosymbionts within the host. The other technique used is Flourescence *in situ* hybridization (FISH) which is a modification of *in situ* hybridization technique (ISH) which is based on formation of Watson-Crick base pairing between the gene of interest and the complementary sequence tagged with a fluorescent reporter molecule. In FISH, a flourophore is tagged to the probe and it acts as the reporter molecule. FISH has been used in the detection and identification of unculturable bacteria from different samples [28,29]. Nucleotide sequences of closely related species can also be differentiated by using FISH [30]. Different types of probes like ssDNA, dsDNA, RNA probes can also be used depending on the target gene of interest. Another kind of probe called Locked Nucleic Acid (LNA) is also being used lately. It has been reported that LNA probes are more sensitive and efficient than DNA probes [31].

Locked nucleic acid (LNA) nucleosides are analogues of nucleic acids consisting of a methylene bridge connecting 2’ O- atom and 4’ C- atom thus locking the ribose ring. The locking of ribose ring provides an ideal confirmation to the LNA nucleosides to show efficient Watson and Crick base pairing. The LNA probes show complementary pairing with DNA or RNA oligonucleotides when put together and increases the stability of duplex formed. The stability is because of increase in the melting temperature of the resulting duplex. The LNA probes are highly specific and have efficient single nucleotide discrimination and are also resistant to endo and exonucleases [32,33]. FISH analysis has been done in *E*. *coli* by using LNA probes [34]. LNA probes can also be used for detection of specific microRNA and other small RNA molecules in tissues. LNA probes have also been used for detection of bacterial endosymbionts in *B*. *tabaci* [35].

Hence, the aim of present study is to make a detailed comparison of the two molecular techniques—PCR and FISH for identification and localization of bacterial endosymbionts in *B*. *tabaci* and also to identify the different endosymbionts present in different locations with respect to their genetic groups.

## Material and Methods

### Ethics Statement

The field studies did not involve endangered or protected species. No specific permissions were required for these locations/activities as the said insect *Bemisia tabaci* is not an endangered or protected species. Its infestation is seen in natural conditions at different locations and its collection does not require any permission or permit from any regulatory authority under the prevalent laws.

### Whitefly collections

**Fig 1:**
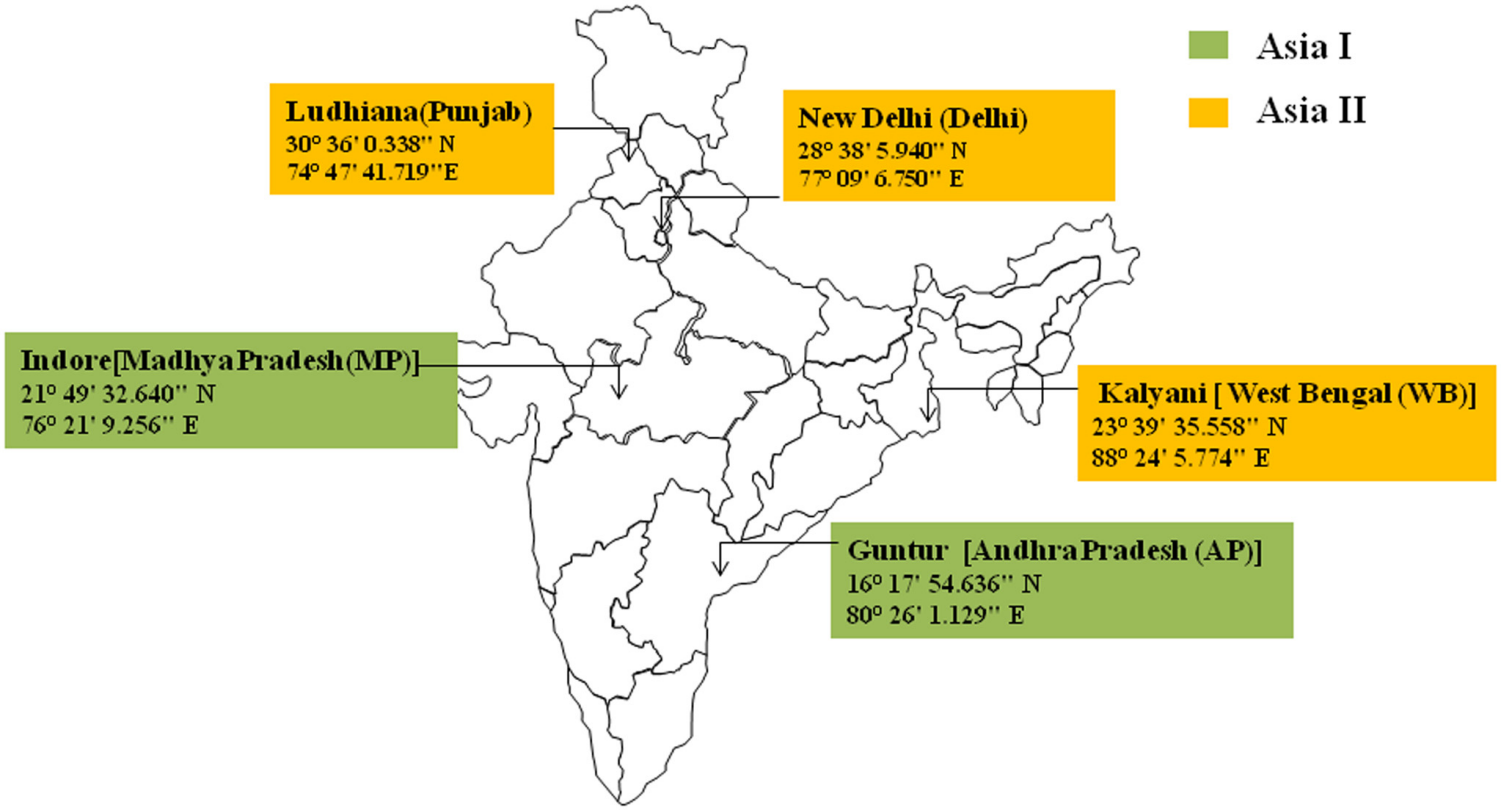
Map showing different locations of whitefly collection. The yellow colour represents Asia II genetic group and green colour represents Asia I genetic group. All the samples were collected from cotton fields.

*B tabaci* samples were collected from different locations in India (Fig 1): New Delhi (Delhi), Ludhiana (Punjab), Guntur (Andhra Pradesh), Kalyani (West Bengal) and Indore (Madhya Pradesh) and reared in insect proof whitefly culture chambers at Indian Agricultural Research Institute (IARI), Pusa, New Delhi, India. The samples were selected randomly with the help of aspirators from abaxial surface of the cotton plant leaves from different chambers. The samples included both males and females. For PCR the samples were collected in 100% ethanol and stored at 4°C while, for FISH analysis the samples were collected in acetone in microcentrifuge tubes and stored at -20°C till further use. The genetic group of whiteflies from different locations was identified according to Singh et al. [36], based on mitochondrial cytochrome oxidase 1 (mtCO1) gene markers.

### DNA extraction from *B*. *tabaci* for diagnostic PCR

Single whitefly was used for DNA isolation. Each whitefly was washed in 200 μL of autoclaved water by centrifugation at 5000 rpm for 5 minutes. The washed whiteflies were then homogenized with the help of hand held homogenizer (Sigma Aldrich, Z359971-1EA) in 14 μL of lysis buffer consisting of 100 mM Tris-Cl pH 8.0, 1% SDS, 100 mM NaCl and 100 mM EDTA pH-8 1%. 2 μL of Proteinase K (0.28 μg/μL; Sigma Aldrich, Catalog no. 39450-01-6) was added in the homogenized mixture and mixed properly. The homogenate was then incubated at 65°C for 45 minutes. After incubation 20 μL of pre-chilled 5 M potassium acetate and 8 μL of 6 M lithium chloride was added in the incubated homogenate and kept in ice for 15 minutes. The mixture was then centrifuged at 10000 rpm for 15 minutes. After centrifugation the supernatant was transferred to fresh microfuge tube and 0.6 volume of isopropanol was added. The supernatant and isopropanol mixture was again centrifuged at 10000 rpm for 15 minutes. The pellet obtained after centrifugation was washed in 70% ethanol. The pellet obtained was air dried and dissolved in elution buffer (10 mM Tris-Cl, pH-8.0). RNase treatment (0.1μg/μL) was given for 45 minutes at 37°C. The DNA was then checked on 0.5% agarose gel containing ethidium bromide (0.5μg/mL). The gel was run at 110 volts for 15 minutes and then observed by UV transilluminator (FOTODYNE incorporated, USA).

### Diagnostic PCR for presence of bacterial endosymbionts in *B*. *tabaci* samples

*B tabaci* population from 5 different locations of Delhi, Punjab, Guntur (Andhra Pradesh), Kalyani (West Bengal) and Indore (Madhya Pradesh) were taken from whitefly culture chambers in IARI, Pusa, New Delhi. 20 whiteflies for each of the five populations were collected randomly for the experiment and diagnosed for the presence of different bacterial endosymbionts- *Portiera*, *Wolbachia*, *Rickettsia*, *Arsenophonus* and *Cardinium*. Specific bacterial primers were used for amplification of 16S rRNA bacterial gene (Table 1). For each bacterial endosymbiont, PCR mix was containing dNTPs (2.5 mM), 1X buffer (2.5 μL), Taq polymerase (1U), Forward and Reverse primers (7.5 pmol each), DNA template (25–30 ng) and the final volume of 25 μL was prepared with autoclaved water.

**Table 1:** Primers and PCR cycling conditions for the identification of bacterial endosymbionts associated with *B*. *tabaci*.

Endosymbiont	Primer sequence	PCR Cycles	Annealing Temperature	Product Size	Reference
*Portiera*	F-5’TGCAAGTCGCGGCATCAT3’	45	58°C	1000bp	[37]
	R-5’CCGCCTTCTGCGTTGGCAACT3’				
*Wolbachia*	F-5’CGGGGGAAAATTTATTGCT3’	45	52°C	650bp	[37]
	R-5’AGCTGTAATACAGAAAGGAAA3’				
*Rickettsia*	F-5’GCTCAGAACGAACGCTGG3’	45	55°C	800bp	[26]
	R-5’GAAGGAAAGCATCTCTGC3’				
*Arsenophonus*	F-5’CGTTTGATGAATTCATAGTCAAA3’	45	52°C	630bp	[37]
	R-5’GGTCCTCCAGTTAGTGTTACCCAAC3’				
*Cardinium*	F-5’GCGGTGTAAAATGAGCTTG3’	45	50°C	440bp	[23]
	R-5’ACCTCTTCTTTAACTCAAGCCT3’				

Denaturation was carried for all bacteria at 94°C for 30 seconds. Annealing was carried out at different temperatures specific for each bacterial endosymbiont (*Portiera* 58°C, *Wolbachia* 52°C, *Arsenophonus* 55°C, *Rickettsia* 55°C, *Cardinium* 50°C) for 30 seconds. Extension was carried out at 72°C for 40 seconds with the final extension for 5 minutes at same temperature. 45 number of cycles were fixed for each bacterial endosymbiont detection.

Both positive and negative controls were used for each reaction. The plasmids containing 16S rRNA gene of different bacterial endosymbiont were used as positive controls while the reaction without any DNA was used as negative control. *Portiera* being the primary endosymbiont was expected to be present in all the samples. The PCR product was then checked on 0.8% agarose gel and the PCR products for different bacteria exhibited bands of different band length (*Portiera* 1000bp, *Wolbachia* 650bp, *Arsenophonus* 630bp, *Rickettsia* 800bp, *Cardinium* 440bp).

### Locked Nucleic Acid (LNA) Probes

The LNA probes used were specific in sequence for specific bacterial endosymbionts. The LNA probes were supplied by Exiqon A/S. The LNA probe sequences for different endosymbionts are given in the Table 2. The concentration of probes used for all the endosymbionts was 10 nmoles per mL.

**Table 2:** LNA probe sequences for different endosymbionts.

Endosymbiont	5’-3’ sequence	Flourescent dye at 5’ end	Product number	Batch number	Reference
*Portiera*	TGTCAGTGTCAGCCCAGAAG	56FAM	500150	503271	[38]
*Wolbachia*	CTTCTGTGAGTACCGTCATTATC	TEX615	500150	503275	[38]
*Arsenophonus*	TCATGACCACAACCTCCAAA	TYE665	500150	503277	[38]
*Rickettsia*	TCCACGTCGCCGTCTTGC	TYE563	500150	503272	[38]
*Cardinium*	TATCAATTGCAGTTCTAGCG	TYE705	500150	503273	[38]

### Flourescent *In situ* Hybridization

The whitefly samples from different locations stored in acetone were processed for FISH analysis. *B*. *tabaci* samples were fixed in the Carnoy’s fixative (Ethanol: Chloroform: Glacial acetic acid, 6:3:1) overnight. The fixed whitefly samples were kept in 6% H_2_O_2_ for 48 hours for decolouration. The decolourized flies were treated with 50 μL of hybridization buffer (20 mM Tris-Cl, pH-8, 1% Sodium dodecyl sulphate, 0.9 M sodium chloride, 30% Formamide) containing specific LNA probes for different bacterial endosymbionts. Different combinations of LNA probes were used depending on the specific flurophores used in the probes. The mixture was then incubated at 42°C overnight. The overnight mixture was taken out and the whitefly samples were washed twice with washing buffer (0.03 M sodium citrate, 0.3 M sodium chloride, 0.01% SDS-sodium dodecyl sulphate) for 15 minutes. The washed whiteflies were then mounted on slides using Vectashield (Vector Labs). For each location 20 replicates for each bacterial endosymbiont were taken. Then the slides were observed for different bacterial endosymbionts on Nikon A1 confocal microscope and images were acquired at fixed camera and microscope settings for LNA probes. NIS elements (V3.21.02) image analysis software (Nikon) was used for quantifying the fluorescence intensities for different bacterial endosymbionts.

## Results

**Fig 2:**
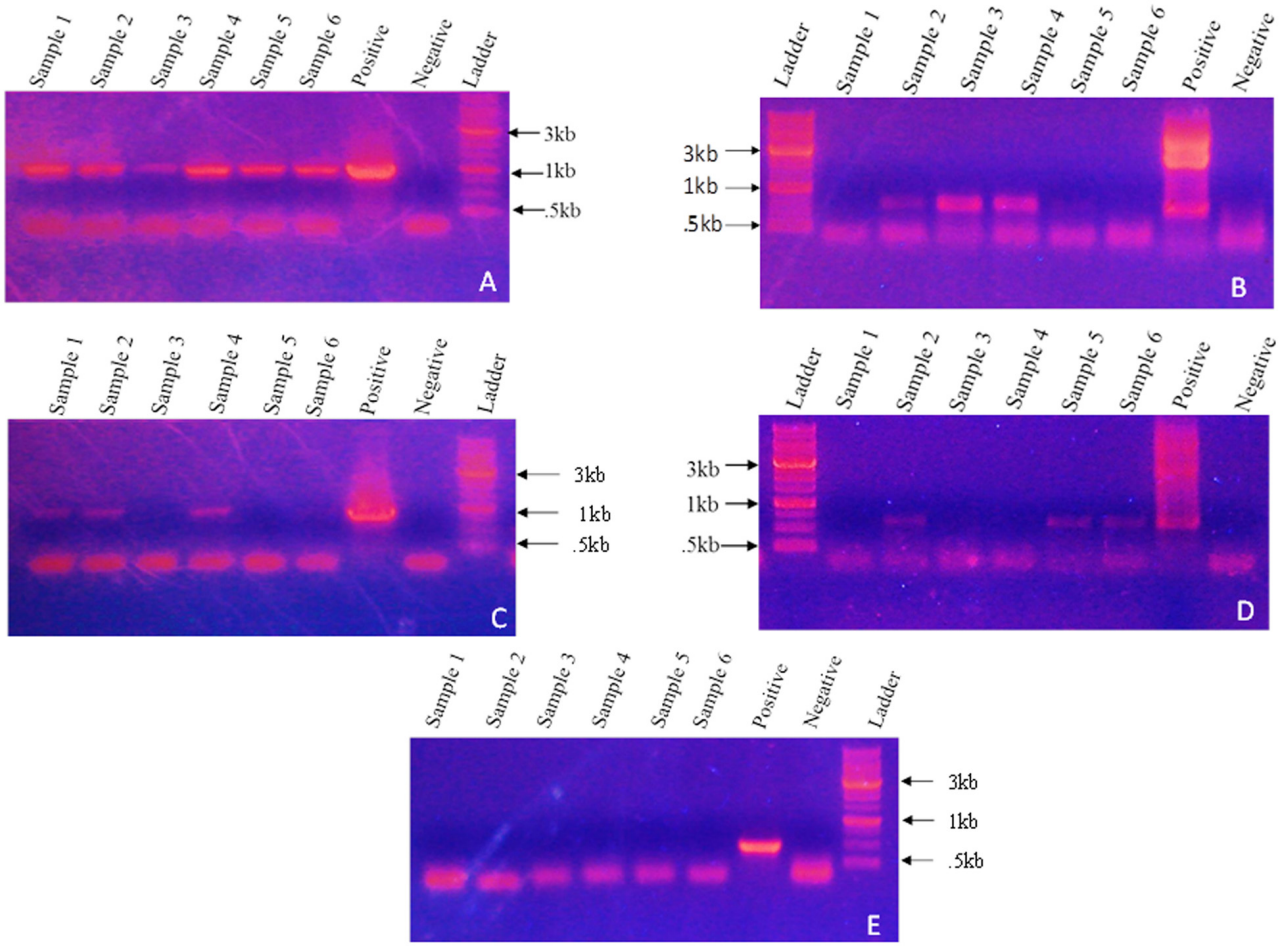
Agarose gel electrophoresis of 16S rDNA PCR product of different bacterial endosymbionts, amplified from total DNA of *B*. *tabaci* samples of Delhi population. **(A)** Represents PCR results for primary endosymbiont *Portiera* (1kb). **(B)** *Wolbachia* (650bp). **(C)** *Rickettsia* (800bp). **(D)** *Arsenophonus* (630bp). **(E)** *Cardinium* (440bp) (Not detected from any of the samples from any location.)

The genetic groups of the *B*. *tabaci* collected from different locations have been shown in Fig 1 and our results show no difference in the structure of endosymbionts from these locations across India. Genomic DNA was isolated from 20 different individuals of each population and used for detection of different endosymbionts by bacteria specific diagnostic PCR, whose results are summarized in Table 3. As a sample, results from 6 individual whitefly of Delhi population is pictorially represented in Fig 2, where in presence of *Portiera* is indicated by an approximate 1000 bp PCR product, presence of *Wolbachia* by 650 bp, *Rickettsia* by 800 bp, *Arsenophonus* by 630 bp and *Cardinium* by a 440 bp PCR product. Positive and negative controls were included in each PCR, which gave the intended results. Analysis of Fig 2 indicates that *Portiera* is present in all the 6 samples, while *Wolbachia*, *Rickettsia* and *Arsenophonus* is present in only some of the 6 whitefly individuals, and *Cardinium* was absent in all of them. The detection of the four secondary endosymbionts was not uniformly detected (positive) in every individual and thus the 20 individuals from each location showed varied infection frequencies (Table 3). This points to the fact that bacteria specific diagnostic PCR does not detect secondary endosymbionts in all the individuals of a population from a particular location.

**Table 3:** Detection of different endosymbionts from different population on basis of 16S gene primer specific diagnostic PCR and FISH analysis by LNA probes.

Total no. of samples for each bacteria for both PCR and FISH separately	Location	*Portiera* present				*Wolbachia* present				*Rickettsia* present				*Arsenophonus* present				*Cardinium* present	
		PCR	FISH	χ^2^	P	PCR	FISH	χ^2^	P	PCR	FISH	χ^2^	P	PCR	FISH	χ^2^	P	PCR	FISH
20	Delhi	19	20	0.05	0.8	12	19	3.25	0.05	9	16	6.13	0.01	9	16	6.85	0.01	0	0
20	Punjab	18	20	0.2	0.7	9	18	6.25	0.01	8	17	7.65	0.01	11	15	5.30	0.01	0	0
20	Guntur (AP)	18	20	0.2	0.7	7	19	8.50	0.01	3	17	14.9	0.001	14	17	2.25	0.1	0	0
20	Kalyani (WB)	18	20	0.2	0.7	5	20	11.25	0.001	0	18	20.20	0.001	6	17	10.45	0.001	0	0
20	Indore (MP)	20	20	-	-	0	17	20.45	0.001	13	14	4.25	0.05	3	18	14.65	0.001	0	0

**Fig 3:**
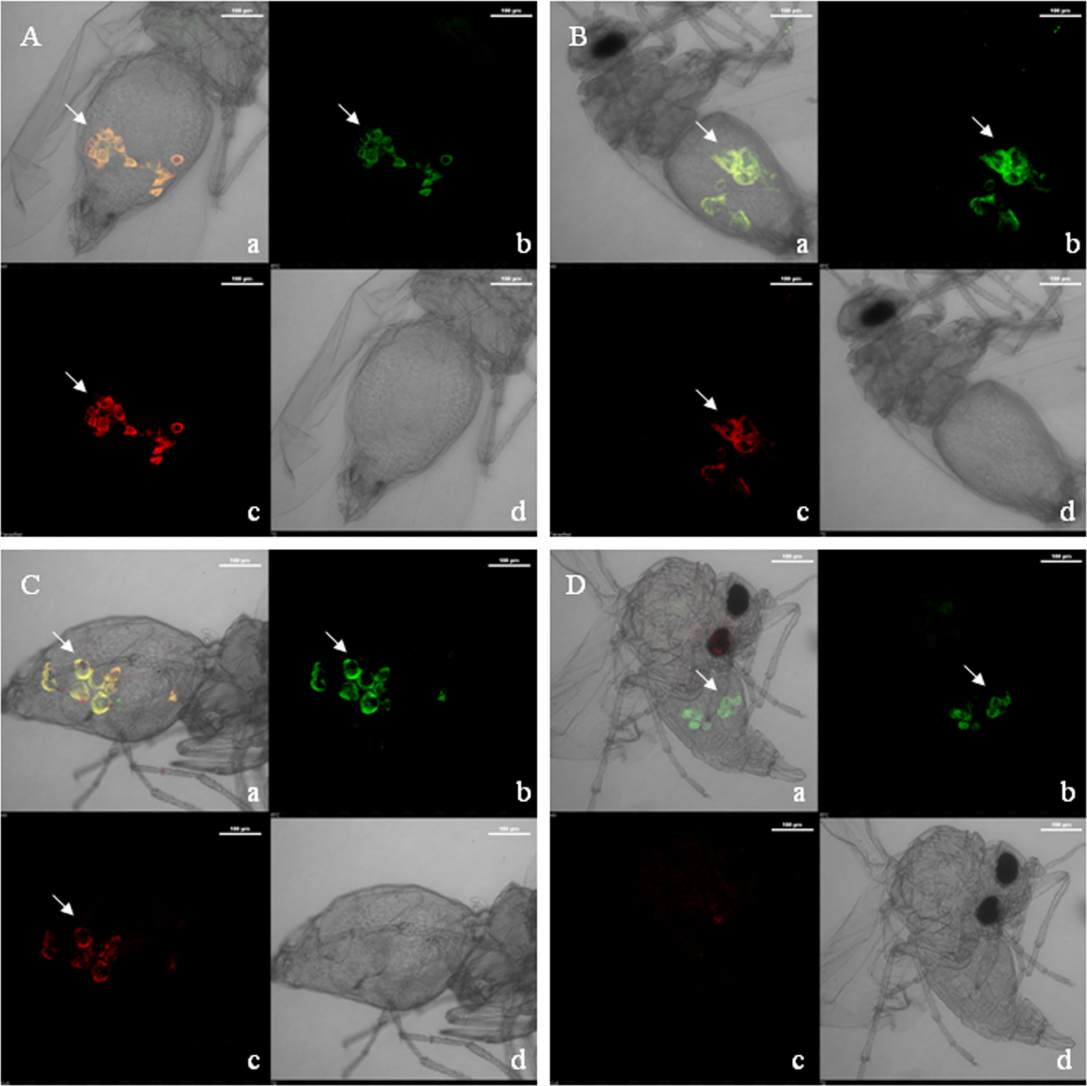
FISH staining of different bacterial endosymbionts in whole mount of *B*. *tabaci* using bacteria specific LNA probes. Arrows indicate the bacteriocytes. *Portiera* is used as a control for other secondary endosymbionts. **(A)** Localization of *Wolbachia* and *Portiera*. **(B)** Localization of *Rickettsia* and *Portiera*. **(C)** Localization of *Arsenophonus* and *Portiera*. **(D)** Localization of *Cardinium* and *Portiera* (*Cardinium* being absent in this case). **(a)** Merged image showing overlap of *Portiera* and *respective secondary endosymbiont*. **(b)** Presence of *Portiera* in bacteriocytes. **(c)** Presence of respective secondary endosymbiont in bacteriocytes **(d)** Phase contrast.

FISH, by using fluorescent DNA oligo is another method to detect endosymbiotic bacteria in insects. Representative results are depicted in Fig 3 of whole mounts of *B*. *tabaci* from Delhi population for each of the bacterial endosymbiont. For detection of each secondary endosymbiont, the detection of *Portiera*, the primary endosymbiont was used as a positive control. Results obtained by FISH indicate that *Portiera* is present in all locations, similar to the results by PCR. It also indicates the presence of *Wolbachia*, *Rickettsia* and *Arsenophonus* in all locations and the total absence of *Cardinium*. The proportion of positive detection by FISH appears to be significantly greater, than that by PCR, for samples from the same population (Table 3).

**Fig 4:**
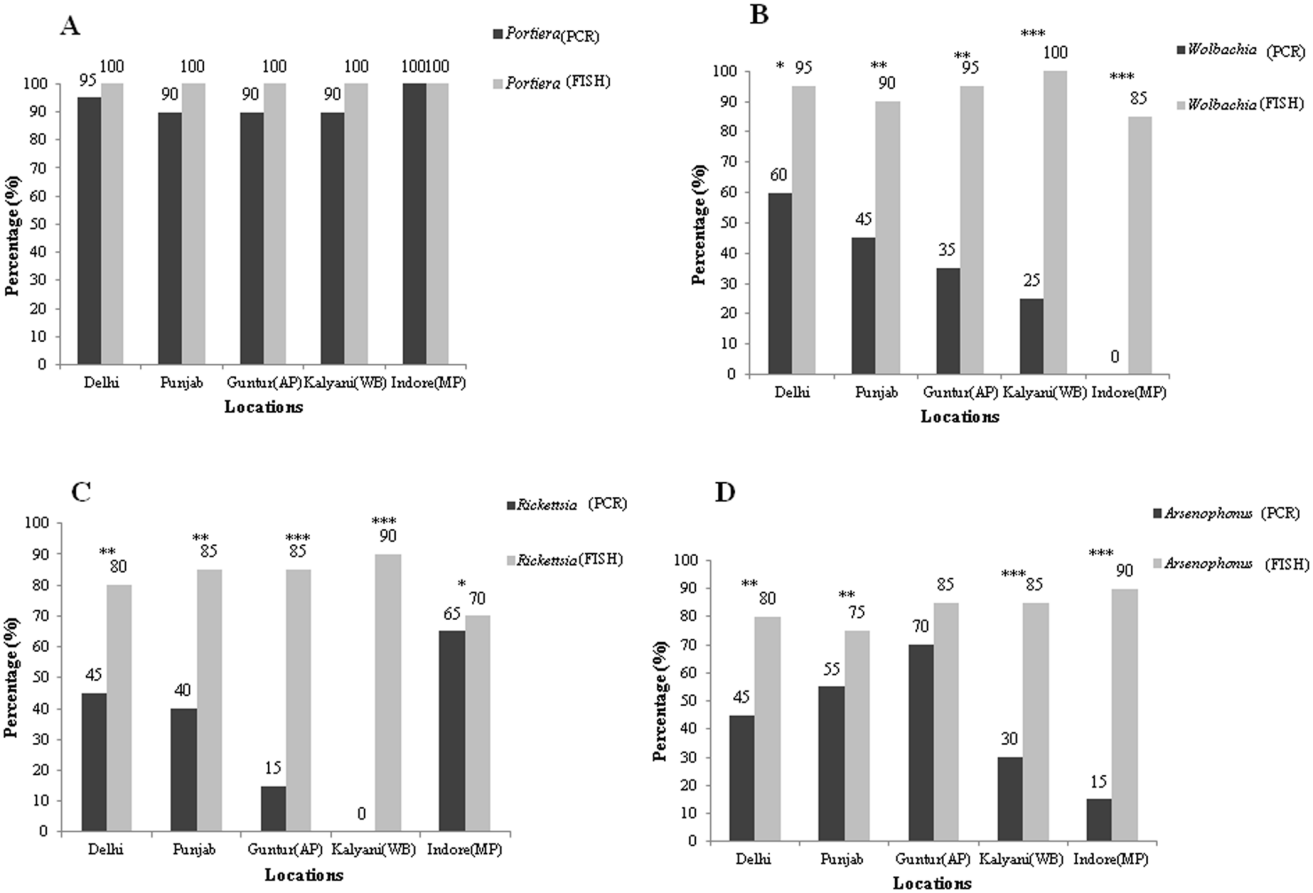
Comparative frequency distribution of different bacterial endosymbionts by diagnostic PCR and FISH in samples of *B*. *tabaci* from different locations. **(A) *Portiera***. On an average, from all locations, *Portiera* was detected in 93% of the samples by PCR and 100% by FISH (χ^2^ = 0.49, P = 0.50). **(B) *Wolbachia***. On an average, from all locations, *Wolbachia* was detected in 33% of the samples by PCR and 93% by FISH (χ^2^ = 45.38, P = 0.001). **(C) *Rickettsia***. From all locations on an average 33% of *Rickettsia* was detected by PCR and 82% by FISH (χ^2^ = 48.13, P = 0.001). **(D) *Arsenophonus***. On an average from all locations 43% of the samples were detected to have *Arsenophonus* by PCR and 83% by FISH (χ^2^ = 35.38, P = 0.001). The values above the bars give the percentage of bacterial endosymbionts in those locations detected respectively by two techniques. The asterisks over bars shows the significant difference based on χ^2^ values for samples from same location for each of the endosymbionts detected by two techniques. *P ≤ 0.05, **P ≤ 0.01, ***P ≤ 0.001.

In order to statistically compare the efficiency of these techniques for detecting bacterial endosymbionts, the results were (i) compared by χ^2^ test and (ii) converted to percentage positive detection, which were then compared for each bacteria in different populations (locations). Fig 4A compares the results obtained by PCR and FISH in detecting *Portiera* in five different locations where in PCR is able to detect *Portiera* in 90–100% of the samples while FISH detects *Portiera* in all the 100 individuals (20 sample × 5 location), but there is no significant difference in the detection abilities between these two techniques. *Portiera* being the predominant primary endosymbiont was detected in all 5 populations and the overall infection frequency of *Portiera* was significantly higher in all populations as compared to other secondary bacterial endosymbionts. All values were non-significant for χ^2^ test (Table 3, Fig 4A). On the contrary, detection of secondary endosymbionts by FISH is significanty superior to detection by PCR. Infection frequency of secondary endosymbiont *Wolbachia*, *Rickettsia* and *Arsenophonus* as detected by PCR was significantly less as compared to detection by FISH with LNA probes. *Wolbachia* could not be detected from Indore population by PCR. In fact, for *Wolbachia* all populations from different locations showed significant differences for χ^2^ test (Table 3, Fig 4B).

Similarly, *Rickettsia* could not be detected from Kalyani (WB) population by PCR. The χ^2^ values for detecting *Rickettsia* either by PCR or by FISH also showed significant differences (Table 3, Fig 4C). Similarly, comparing *Arsenophonus* detection by PCR and FISH from different populations, we found that, except Guntur, the other four locations showed significant χ^2^ values (Table 3, Fig 4D). The percent infection frequency of different bacterial endosymbionts as detected by both techniques from different populations is also presented in Fig 4. Thus, these results compel us to conclude that FISH is a superior technique to detect endosymbiotic bacteria from insect samples.

## Discussion

Detection of bacteria in insects is of major importance for understanding the benefits or losses accounted by these bacteria to the host species. These interactions between bacterial endosymbionts and their hosts are important for host’s ecology, evolution and fitness. In this study, the two techniques viz PCR and FISH have showed varied sensitivity in the detection of bacterial endosymbionts in the insect *B*. *tabaci*. Such comparative studies between the two techniques have been done earlier for detection of translocations in lymphomas [39,40], detection of aneuploidies in single blastomeres [41], detection of tumors in processed tissue [42], identification of different bacteria from patients with cystic fibrosis [43] etc. Some of these studies have clearly considered FISH as superior to PCR [41,42] while others have just compared them. Although, PCR and FISH have been used for the detection of bacterial endosymbionts in insects including aphids, mealybugs, whiteflies etc [26,37,38,44,45,46,47,48,49], no such comparative analysis between the two has been performed. Hence, in view of non-availability of such an account in insects, we comparatively evaluated the sensitivity and applicability of these techniques in detecting bacterial endosymbionts in the insect, *B*. *tabaci*.

In our study, PCR resulted in a lower sensitivity and varied infection frequency while detecting bacterial endosymbionts, which is in accordance with earlier studies [24,38,50]. The low detection by PCR in our study could be because of the actual absence of the endosymbiont in the whitefly sample. On the contrary, when whitefly samples from locations testing negative for a particular endosymbiont through PCR were subjected to FISH, these endosymbiotic bacteria could be detected, thus indicating that endosymbionts are actually present in these samples, but could not be detected by PCR. Another reason for non- detection could be the insensitivity of the PCR protocol to detect lower titres of endosymbionts in the whitefly sample [51]. In fact, it has been suggested that the bacterial community which make up atleast 1% of the total bacterial population in the host can only be detected by PCR [52]. The bacterial endosymbiont population could be lesser than this (1%) level and hence not detected by PCR in our experiments.

It has also been reported that the number of bacteriocytes present in males are less when compared to females and this could also be a reason for the non- detection of endosymbionts in some of our samples by PCR. However, the FISH results obtained with both males and females confirmed high sensitivity of LNA probes as compared to PCR. Moreover, in our earlier study, we have also concluded that the use of LNA probes substantially improve the detection of bacterial endosymbionts by FISH [35].

In Conclusion, our results in this study clearly indicates the acuteness of FISH over PCR in detecting endosymbionts in insect *B*. *tabaci*, which is validated by (a) the increased number of endosymbiont bearing whitefly individuals detected by FISH vis a vis PCR, (b) the increased efficiency of FISH in comparing the infection frequencies both within a population and also among the six different locations irrespective of the genetic group of whiteflies, (c) consistency of the results obtained by FISH in samples from all the locations than obtained by PCR, (d) the increased average presence of secondary endosymbionts determined from all the locations by FISH than that determined by PCR.

In fact, the present data clearly represents the pros and cons of both the techniques and also gives an idea about the possible problems encountered by using these techniques. While on one hand, PCR can only be used for the detection of the endosymbionts, FISH with LNA probes can also be used for localization of the endosymbionts within the host besides detecting them. However, the FISH based detection technique is quite expensive and the probes used are susceptible to rapid freeze and thaw which can affect the quality of signals and consequently the results. Their usage also demands high level of care and precision, with excessive amount of probes leading to non-specific signals. Also, there are chances of obtaining autofluorosence because of probes interacting with fat bodies or some other non-specific structures in the insect body, thus giving false positive results. However, the issue of false positives in FISH can be taken care of by processing the samples without probes and then comparing with the samples processed with probes. This will give an idea about whether the signal obtained in case of samples with probes are really genuine signals for the bacteria or some false signals generated by excitation of some impurities or insect chitinous structure.

Nevertheless, the results obtained from both techniques also revealed the advantages of one over the other. PCR results did not show the presence of some endosymbionts in all the samples but in case of FISH even low intensity signals were detected. In addition, the PCR based detection is a two step process, involving the isolation of DNA from the samples followed by amplification using PCR. If the DNA would be isolated properly, only then the PCR results could be obtained and there are chances of getting non-specific amplification. But in case of FISH, there is no need for DNA isolation which further reduces the chances of error. Also, large number of insect samples can be easily processed simultaneously in FISH as compared to PCR which is more laborious. Moreover, PCR based detection of endosymbionts can be further improved by use of modified bacteria specific primers which can help in detection of even smaller quantities of endosymbionts. Thus, our results clearly give an edge to FISH technique over the PCR and there is an urgent need for more research to be conducted on intelligent usage of different techniques for identification and localization of bacterial endosymbionts in insect species.
